# A comparison of the Allplex™ bacterial and viral assays to conventional methods for detection of gastroenteritis agents

**DOI:** 10.1186/s13104-018-3645-6

**Published:** 2018-07-28

**Authors:** Kelly Amrud, Robert Slinger, Nadia Sant, Marc Desjardins, Baldwin Toye

**Affiliations:** 1 Eastern Ontario Regional Laboratory Association, 401 Smyth Rd., Ottawa, ON K1H 8L Canada; 2 Children’s Hospital of Eastern Ontario, Ottawa, ON Canada; 3 The Ottawa Hospital, Ottawa, ON Canada

**Keywords:** Gastroenteritis, PCR, Viruses, Bacteria, Diagnosis

## Abstract

**Objective:**

Molecular methods to detect diarrheal pathogens are increasingly being used in place of conventional methods. We compared a new multiplex real-time PCR assay for detection of both bacterial and viral gastroenteritis agents, the Allplex™ Gastrointestinal Panel Assays (AGPA), to conventional methods (stool culture for bacterial pathogens and electron microscopy (EM) for viral pathogens).

**Results:**

Gastrointestinal viruses, in particular norovirus genogroup II viruses, were detected by the AGPA in a high number of specimens that were negative by EM. For bacterial pathogens, the AGPA was able to detect the organisms grown in culture with high sensitivity and additionally detected several types of *E. coli*, such as enteropathogenic *E. coli* (EPEC), enteroaggregative *E. coli* (EAEC), and non-O157 Shiga toxin-producing *E. coli* (STEC), that could not be detected with conventional culture methods. Overall, the AGPA had a > 2-fold higher detection rate than the conventional methods, with 24/135 (17.8%) samples positive by conventional methods and 60/135 (44.4%) by AGPA. Thus, diarrhea pathogen detection rates increased substantially with the use of the AGPA as compared to conventional methods.

## Introduction

Detection of gastroenteritis pathogens by molecular methods is becoming more widespread. These methods provide more rapid results than conventional methods such as culture, and allow certain pathogens to be detected for which conventional methods are insensitive or not available [1].

The Allplex™ Gastrointestinal Panel Assays (AGPA) (Seegene, Seoul, South Korea) is a new multiplex real-time PCR assay that detects 13 bacteria, 6 viruses, and 6 parasites in 4 multiplex PCR reactions (two bacterial, one viral and one parasitic). We assessed the performance of the bacterial and viral AGPA in comparison to the conventional methods of bacterial culture and stool electron microscopy (EM) for virus detection from stool samples in our region.

Of note, in our area, the eastern region of the province of Ontario, Canada, the most common bacterial gastroenteritis pathogens detected by conventional culture methods have been *Salmonella* spp., and *Campylobacter* spp. [2]. Viral gastroenteritis has not been studied on a regional level, but, as in the rest of Canada, noroviruses and rotaviruses have historically been the major pathogens [3]. In recent years, with the introduction of universal, publicly-funded rotavirus immunization program in 2011 in the province of Ontario, hospitalizations for rotavirus have decreased significantly [4].

## Main text

### Methods

#### Specimens

This study was performed at the Children’s Hospital of Eastern Ontario (CHEO) and The Ottawa Hospital, Ottawa, ON Canada. Consecutive stool samples that were submitted for both bacterial and viral testing were included in the study. Residual stool samples submitted to the laboratory between January and July 2017 were used for AGPA testing.

In addition, since the number of samples positive for bacterial pathogens in the prospective study was anticipated to be relatively small, a group of archived bacterial culture-positive samples collected prior to the study period was also tested with the AGPA.

#### Conventional diagnostic methods

Stools for bacterial culture were collected in enteric transport medium (modified Cary-Blair medium) and cultured using selective and differential media, with 10 µL of stool used in each media, following standard procedures. Pediatric stool specimens were also cultured on blood agar plate (BA) for *Aeromonas* spp., *Vibrio* spp. and *Plesiomonas* spp. Organisms were identified using standard laboratory methods [5]. A portion of the samples were also tested for *Clostridium difficile* using an enzyme immunoassay for glutamate dehydrogenase (GDH) of *C. difficile* (*C. DIFF CHEK*™—*60*, TECHLAB). If positive for GDH, a Toxin B PCR test (Simplexa™ C. difficile Universal Direct, Focus Diagnostics) was performed. Electron microscopy was used to determine the presence of viruses in stool using standard methods with a JEM 1010 Electron Microscope [6].

#### Molecular diagnostic methods

Stool nucleic acid samples were extracted as recommended by the manufacturer. Briefly, 300 µL stool was added to 1 mL ASL Stool Lysis Buffer (Qiagen, Hilden, Germany) to create a sample suspension. After centrifugation 400 µL of the supernatant was extracted using the MagNA Pure Compact System (Roche Molecular Systems) with a final elution volume of 100 µL. The AGPA multiplex PCR was performed following manufacturer’s recommendations on a CFX96 Real Time Detection System (Bio-Rad, Hercules, CA, USA). One AGPA multiplex panel tested for *Shigella* spp./enteroinvasive *E. coli*, *Campylobacter* spp., *Yersinia enterocolitica*, *Vibrio* spp., *C. C. difficile* toxin B, *Aeromonas* spp. and *Salmonella* spp. The second panel tested for Shiga toxin producing *E. coli* (STEC)/enterohaemorrhagic *E. coli* (EHEC)/enteroinvasive *E. coli* (EIEC), *E. coli* O157, enteropathogenic *E. coli* (EPEC), enterotoxigenic *E. coli* (ETEC), enteroaggregative *E. coli* (EAEC) and hypervirulent *C. difficile*. The viral multiplex panel tested for six viruses: norovirus genogroups I and II (GI/GII), rotavirus A, adenovirus F (Serotype 40/41), astrovirus, and sapovirus.

#### Discrepant analysis

For discrepant organisms detected by the AGPA but not by the conventional tests, we performed monoplex 5′exonuclease probe PCR assays [2, 7, 8]. Results were considered to be true positives if positive by conventional methods or if both the AGPA and monoplex PCR assays were positive. The McNemar test, a statistical method used to compare results for paired samples, was used to analyse differences between AGPA and conventional methods [9].

### Results

The AGPA detected 46/48 (96%) pathogens in the archived culture-positive stool samples. By organism, 26/27 *Salmonella* spp., 9/9 *Campylobacter* spp., 6/6 *Shigella* spp., 4/4 *E. coli* 0157, and 1/1 *Aeromonas* spp. were detected by AGPA. As well, one archived sample grew *Plesiomonas shigelloides*, an organism not included in the AGPA.

In the prospective study, 135 samples were studied, 70 from adults and 65 from children. Table 1 shows the combined study results by specimen for the AGPA as compared to the conventional methods. As shown, 24/135 (17.8%) samples were positive by conventional methods and 60/135 (44.4%) by AGPA.

**Table 1:** Specimen status (≥ 1 bacterial or viral pathogen detected in specimen) for Allplex™ Gastrointestinal Panel Assays (AGPA) and conventional methods

	Conventional methods positive	Conventional methods negative	Totals
AGPA positive	23	37	60
AGPA negative	1	74	75
Totals	24	111	135

There were 38 samples with discrepant results, 37 AGPA-positive, conventional method-negative samples, and 1 conventional methods-positive, AGPA-negative sample. Monoplex PCR assay results confirmed the AGPA results for 33/37 (89.2%) AGPA-positive discrepant samples. Thus, after discrepant sample analysis, there were 33 true-positive samples detected by AGPA but not by conventional methods, and 1 true-positive sample detected by conventional methods only. This difference was statistically significant (p < 0.001).

Results by pathogen detected are shown in Table 2. The proportion of samples that tested positive for more than one target by the three AGPA assays was 14/60 (23.3%). For example, norovirus genogroup (G) II was detected with other agents in 7/26 (26.9%) specimens. Six of these samples had two pathogens identified. The other organisms detected along with norovirus GII in these 6 samples were: *C. difficile* (in 2 specimens), EPEC, adenovirus, *Campylobacter* spp., and shiga toxin producing *E. coli* (non O157). The sample that contained three pathogens was positive for norovirus GII, astrovirus, and EAEC.

**Table 2:** Number of pathogens detected by conventional methods and Allplex™ Gastrointestinal Panel Assays in the prospective study of 135 fecal specimens

	Conventional methods	Allplex assays
Bacteria		
*Salmonella* spp.	1	2
*Shigella* spp.^a^	1	1
*Campylobacter* spp.	4	5
*Yersinia enterocolitica*	1	2
*Clostridium difficile* toxin B^a^	9	15
*Aeromonas* spp.^b^	0	3
Vibrio spp.^b^	0	0
*E. coli* O157	0	1
Shiga toxin producing *E. coli* (non-*E. coli* O157)	NA	2
Enteropathogenic *E. coli*	NA	5
Enterotoxigenic *E. coli*	NA	0
Enteroaggregative *E. coli*	NA	3
Hypervirulent *Clostridium difficile*	NA	1
Viruses		
Norovirus GII	5^c^	26
Norovirus GI	0	0
Rotavirus	2	4
Adenovirus	0	1
Sapovirus	1^c^	3
Astrovirus	0	2
Small round virus	1	0

^a^Only 91/135 samples were tested for *C. difficile* using conventional methods

^b^Only 65/135 samples were tested for *Aeromonas* and *Vibrio* spp. using conventional methods

^c^Identified as “small round viruses” by EM

### Discussion

Overall, the AGPA detected over twofold more bacterial and viral pathogens than the conventional methods in the prospective study, and also was able to detect a high proportion of the bacteria in the archived culture-positive samples. The AGPA method also enabled detection of diarrheagenic *E. coli* strains for which culture media are not available, such as EPEC, EAEC, and non-*E. coli* O157 Shiga toxin producing strains.

Detection of bacterial pathogens by AGPA was also faster than conventional methods, requiring approximately 4 h vs the 24–72 h required for bacterial culture and identification.

Similar results showing greater detection rates with use of the AGPA method have been shown for viruses [10] and bacteria [11]. In a study comparing the AGPA viral panel to another multiplex PCR assay (the Seeplex Diarrhea-V Ace Detection), the overall agreement was > 90% for the two assays. In addition, the AGPA was also able to detect sapoviruses, which could not be detected with the other assay [10]. In a study of the AGPA bacterial panels conducted in Spain, conventional methods detected a pathogen in 27.7% of specimens. In contrast, the AGPA detected a pathogen in 66.2% of specimens. Looking at the results for the AGPA bacterial panels alone in our prospective study, bacterial pathogens were detected in 16/135 (11.9%) specimens by conventional methods and 34/135 (25.2%) by AGPA. In both our study and the published study, AGPA detected > 2-fold more positive specimens than conventional methods. (The higher rates of detection in the Spanish study may be due to geographic differences in the risks of gastrointestinal bacterial infections.)

## Limitations

A limitation of our study was the use of EM as the comparator method for viral detection in stool samples. As we observed in this study, EM has been found to be less sensitive than molecular methods for diagnosis of viral gastroenteritis [12], so use of lab-developed or commercial molecular methods as the comparator would have been preferable. However, EM was the method in use for viral testing of stool samples at our Virology laboratory at the time of the study. A second limitation of the use EM was that viruses such as noroviruses, astroviruses, and sapoviruses could not be differentiated with this method, and were reported as “small round viruses”.

There are also limitations of the AGPA method. Detection of pathogen nucleic acids may be due to the presence of non-viable rather than live organisms. As well, the need to perform antibiotic susceptibility testing for some bacterial pathogens will require that culture be performed on some AGPA-positive specimens. Another limitation is the inability of AGPA to distinguish *Shigella* spp. from enteroinvasive *E. coli*. In addition, certain organisms such as *Plesiomonas shigelloides* are not included in the panel, but can be detected by culture. Finally, a high proportion of samples with ≥ 1 pathogen were seen with use of the AGPA. Reporting of multiple organisms may create uncertainty for clinicians as to the true cause of the gastroenteritis.

In conclusion, the AGPA method detected significantly more viral and bacterial pathogens than the conventional comparator methods. Future studies should examine the clinical impact of the use of the AGPA method to determine if faster and more comprehensive pathogen detection leads to improvements in patient care.

## Abbreviations

AGPA: Allplex™ Gastrointestinal Panel Assays
EM: electron microscopy
PCR: polymerase chain reaction
EPEC: enteropathogenic *E. coli*
EAEC: enteroaggregative *E. coli*
STEC: Shiga toxin-producing *E. coli*
EHEC: enterohaemorrhagic *E. coli* (EHEC)
GDH: glutamate dehydrogenase
